# A database of eels and their freshwater habitats in southwestern Europe

**DOI:** 10.1038/s41597-026-07213-3

**Published:** 2026-05-09

**Authors:** Maria Mateo, Cédric Briand, Maria Korta, Laurent Beaulaton, Hilaire Drouineau, Hervé Pella, Elsa Amilhat, Agnès Bardonnet, Carlos Antunes, Ramón De Miguel Rubio, Isabel Domingos, Carlos Fernández-Delgado, Mathilde Labedan, Mercedes Herrera, Pierre Sagnes, Lluis Zamora, Estibaliz Díaz

**Affiliations:** 1 Sustainable Fisheries Management, AZTI, Sukarrieta, 48395 Spain; 2 Eaux & Vilaine, Redon, 35600 France; 3 Management of Diadromous Species in their Environment, OFB-INRAE-Institut Agro-UPPA, Rennes, 35000 France; 4 Service for Conservation and Sustainable Management of Exploited Species, SGEEX, OFB, Rennes, 35000 France; 5 Aquatic Ecosystems and Global Change, EABX, INRAE, Cestas, 33612 France; 6 RiverLy, INRAE, Villeurbanne, 69100 France; 7 Centre for Training and Research on Mediterranean Environments, UMR 5110, CNRS - University of Perpignan Via Domitia, Perpignan, F-66100 France; 8 Behavioral Ecology and Fish Population Biology, ECOBIOP, Aquapôle, INRAE-University of Pau and Pays d’Adour, St Pée sur Nivelle, 64310 France; 9 Interdisciplinary Centre of Marine and Environmental Research, CIIMAR-University of Porto, 4450-208 Matosinhos, Portugal; 10 Department of Zoology, University of Córdoba, Córdoba, 14080 Spain; 11 MARE, Marine and Environmental Sciences Centre/ARNET-Aquatic Research Network, University of Lisbon, 1749-016 Lisbon, Portugal; 12 Department of Animal Biology, Faculty of Sciences, University of Lisbon, 1749-016 Lisbon, Portugal; 13 Ecohydraulics Research and Development Unit, OFB-IMFT-PPRIME, OFB, 31400 Toulouse, France; 14 GRECO, Institute of Aquatic Ecology, University of Girona, Girona, 17003 Spain

**Keywords:** Freshwater ecology, Environmental impact, Hydrology

## Abstract

The European eel stock (*Anguilla anguilla*) is outside safe biological limits. A range-wide stock assessment requires the creation and standardisation of databases that include information on eels and their habitats in different countries throughout their distribution range. The SUDOANG 1.0.4 database compiles standardised data on river courses in France and the Iberian Peninsula (Spain and Portugal). Using GIS tools, information on water surface and on other potential aquatic habitats surrounding each river segment has been collected. This common river network provides tools to quickly accumulate information along the river or along the natural path of migration from/to the sea. The database also compiles information on the surface of other habitats, human pressures (including 106400 obstacles), and provides eel abundance and biometric estimations derived from the Eel Density Analysis (EDA) model at the river reach scale for the reference year 2015. The river network supports ecological assessment of the eel habitats, and should also be useful for studies on other migratory species.

## Background & Summary

The European eel (*Anguilla anguilla*) is a catadromous species that spawns at sea and grows in continental waters from northern Africa to Norway^1^, in a wide range of fresh and salt water habitats. Reproduction takes place in the Sargasso Sea^2,3^. Leptocephali larvae undergo a long oceanic drift until they reach the continental shelf, where they metamorphose into glass eels (transparent young eels)^1^. Then, they enter continental waters, where they grow and become pigmented yellow eels. After a growth phase lasting from two to about 25 years^4^, yellow eels become silver eels, at which stage they migrate back to the sea to undertake maturation. The European eel is a semelparous fish species, reproducing only once during its life cycle^5^.

Its stock is outside safe biological limits^6^ and is classified as critically endangered by the International Union for Conservation of Nature (IUCN)^7^. The European eel is threatened by different components of global change^8^, including changes in oceanic conditions^9^ resulting from global warming, overfishing^10^, introduction of an alien roundworm that parasitizes the swim bladder^11^, habitat loss due to fragmentation by artificial obstacles^12^, and contamination^13^. As a consequence, the European Union established in 2007 the Eel Regulation (EC) No. 1100/2007, which obliges all Member States to implement Management Plans for European eel recovery. Moreover, Member States are required to monitor and report several indicators related to stock biomass (or escapement, defined as the quantity of silver eel migrating back to sea) and human-induced mortality in their national waters. However, the population has shown no signs of recovery since then^4^. Eel stock management is hampered by the lack of consistent biomass estimates. The stock is widely distributed across different habitats and jurisdictions, which makes assessment particularly challenging. Moreover, stock indicators are currently obtained using different methodologies depending on the country and even the region, and in many cases they are based on blind extrapolations from other unrelated regions^14^.

The SUDOANG project (acronym resulting from the combination of SUDOE, an interregional EU-funded program for southwest Europe, and ANGuilla, see https://sudoang.eu/en/) has provided managers with common tools and assessment methods to support eel conservation in the SUDOE zone (southern France and the Iberian Peninsula). One of the main project goals was to develop an eel abundance (total numbers), density (kg/ha) and distribution atlas in the three countries, based on the results of the Eel Density Analysis (EDA) model^15–17^. EDA extrapolates eel densities from a range of river segments sampled by electrofishing to the entire basin by accounting for how eel density, size, and sex vary with habitat-related parameters. Thus, EDA requires two main data sources: (i) river characteristics as well as characteristics of other possible aquatic habitat surrounding each river segment, and (ii) eel abundance and biometric data. However, this type of information is sourced from diverse agencies and programs using heterogeneous data formats, including Water Framework Directive (WFD), Community Framework for the Collection, Management and use of data in the fisheries sector (EUMAP), Eel Management Plans (EMP), research groups, scientific papers, and technical reports. Therefore, the first step towards estimating eel densities over a broad geographic area is to compile a common and standardised database of rivers and other aquatic habitats surrounding river segments and its associated eel data coming from both field collections and model outputs. This database covers the Iberian Peninsula and extends to the whole of France (not only the SUDOE zone).

The extensive efforts invested in data collection, cleaning, and standardisation, as well as eel-related parameter estimations produced by EDA, have yielded valuable insights. These fruits should be shared widely to advance both conservation management and ecological understanding. Moreover, beyond a large application for eel, the habitat information gathered is relevant to stakeholders engaged in river management and research, since information on freshwater networks is essential for the conservation and management of aquatic habitats and the species inhabiting them.

## Methods

The SUDOANG 1.0.4 database compiles data on rivers and adjacent water bodies (estuaries, lagoons, reservoirs, lakes, and temporary lakes), as well as physical characteristics and eel data, the latter derived from both field collections and model outputs, across France and the Iberian Peninsula. Mapping of the hydrographic network was based on river nodes and river segments. There are three types of river nodes: points of river confluence (where rivers meet or split), river sources, and river outlets. These outlets can be either at the sea (river mouth) or inland, in the case of endorheic rivers. River segments are represented as irregular lines corresponding to river reaches between nodes. Each river segment is associated with a unit drainage basin, defined as a polygon surrounding the segment. The database is further complemented with adjacent water body polygons describing large or wide aquatic habitats (e.g. estuaries, lagoons, reservoirs). Taken together, the unit drainage basins and the adjacent water body polygons cover the entire catchment, thereby describing the surface area of additional aquatic habitats surrounding the river network. The database comprises three tables: the River Network (RN) table, which contains river maps; the River Network Attribute (RNA) table, which includes segment-level features; and the River Network Eel (RNE) table, which provides information on eel density and distribution. The SUDOANG 1.0.4 database corresponds to the Eel Atlas published on Zenodo (10.5281/zenodo.7546419)^18,19^. Additional EDA input datasets are published at 10.5281/zenodo.8348353(raw electrofishing data for the Iberian Peninsula)^20^, 10.5281/zenodo.6397009(eel data and associated environmental data across the three countries)^21^, and 10.5281/zenodo.8348374(data on dam impacts and cumulative pressures)^22^.

### Data sources

The external data sources used to build and harmonise the SUDOANG 1.0.4 database, providing information on rivers and other water bodies, artificial obstacles, and eels (through electrofishing surveys), are listed below:

- INSPIRE Hydrography (European Directive 2007/2/EC).
- Theoretical Hydrographic Network (RHT, France)^23^.
- BD TOPAGE® (France water bodies)^24^.
- MERIT Hydro database: global hydrography dataset. License, CC-BY-NC 4.0^25^.
- River and Catchment of Europe - CCM^26^.
- HydroATLAS^27^.
- Referential of Flow Obstacles (ROE, France)^28^.
- Information on Ecological Continuity (ICE, France)^29^.
- Flow Obstruction Database (BDOE, France).
- ASPE database (France)^30^.
- RSA database (France).

### River network (RN table)

The hydrographic network of France and the Iberian Peninsula, compiled in the RN table, provides the topological foundation for the EDA model. EDA 2.0 version was based on the Catchment Characterisation and Modelling (CCM), a database that contains information on rivers at the European scale^26^. However, the CCM excludes headwaters and therefore leaves out a substantial part of eel habitat, leading to an underestimation of eel abundance. Therefore, the latest EDA implementation (EDA 2.3) was based on a network that, in addition to covering different countries, has a sufficient spatial resolution to include most of the eel’s habitat, with the exception of some of the largest French estuaries. The European Directive 2007/2/EC for the Infrastructure for Spatial Information in the European Community (INSPIRE) aims to ensure compatibility and interoperability of spatial data infrastructures across Member States, especially in cross-border contexts. The river network developed under INSPIRE contains harmonized layers and includes almost all potential eel habitat. For that reason, we used them in Spain^31^ and Portugal^32^. Note that in Spain, the INSPIRE river network has been generated from elevation data with no field validation of the effective existence of smallest rivers. We removed all estimated small rivers segments not visible during visual checks. In France, we used the Theoretical Hydrographic Network (RHT)^23^, a simplified version of BD CARTHAGE^33^. This network includes very useful additional information not provided by INSPIRE for the implementation of the models used in SUDOANG: a fully chained (*i.e*. each reach is explicitly connected to downstream and/or upstream reaches) and widely used network^34,35^, and most importantly the estimated flow, which in turn allows the modelling of flow through dams and the estimation of turbine mortality^36^. The RN table describes river topography, including segments, upstream and downstream nodes, information on the code of the next downstream segment, and whether the segment flows into the estuary to meet the sea, corresponds to a source, or belongs to an endorheic river (Table 1). The Eel Atlas incorporates some spatial tools, such as a calculator to measure the distance between two river segments along the river network, by summing the lengths of all intervening segments between their upstream and downstream nodes^18,19^.

**Table 1:** Name and description of the topological and hydrographic characteristics recorded for each river segment in France and the Iberian Peninsula, stored in the River Network (RN) table.

Name	Description
Identifier of the river segment (*idsegment*)	Unique identifier of each river segment, starting with the country code.
Upstream node of the river segment (*source*)	Identifier of the immediate upstream node of the river segment; it represents segment-level connectivity and only coincides with the river source for upstreammost reaches.
Downstream node of the river segment (*target*)	Identifier of the immediate downstream node of the river segment; it represents segment-level downstream connectivity.
Length of the river segment (m) (*lengthm*)	Length of the river segment measured along the segment geometry (following its twists and turns) between the upstream and downstream nodes, in meters.
Identifier of the next downstream river segment (*nextdownidsegment*)	Identifier of the immediate and unique downstream river segment connected to the current river segment. This field is null for sea segments (*issea = TRUE*) and may refer to a segment in another country for frontier segments (*isfrontier = TRUE)*.
Chain of segments from the sea to the current river segment (*path*)	Ordered chain (*i.e.* succession of river segments) from the sea to the current river segment.
Is the river segment an international boundary segment? (*isfrontier*)	Indicates whether the downstream node of the river segment is located at an international boundary, where river nodes were defined to ensure connectivity across countries.
Is the river segment a source? (*issource*)	Indicates whether the river segment corresponds to a hydrological river source, *i.e*. it has no upstream river segments (*issource = TRUE*).
Identifier of the sea segment (*seaidsegment*)	Identifier of the most downstream river segment, corresponding to the segment flowing into the sea or into transitional waters (e.g. estuaries). This field is null for endorheic rivers.
Is the river segment flowing into the estuary to meet the sea? (*issea*)	Indicates whether the river segment is an outlet of the river network, i.e. it has no downstream river segments and corresponds to the downstream end flowing into the sea or into transitional waters (e.g. estuaries) (issea = TRUE).
Is the river segment endorheic? (*isendoreic*)	Indicates whether the river segment is part of an endorheic river, *i.e*. not flowing into the estuary to meet the sea (*isendoreic = TRUE*).
Is the river segment part of an international catchment? (*isinternational*)	Indicates whether the river segment is part of an international basin (*i.e*. a basin with river segments located in different countries) (*isinternational = TRUE*).
Country code (*country*)	Code of the country to which the river segment belongs: Spain (SP), France (FR) and Portugal (PT).
Geometry of the river segment (*geom*)	The geometry (*i.e*. PostGIS spatial data type used to represent spatial objects) of the river segment, using the coordinate system EPSG:3035, type MULTILINESTRING.

Considerable effort was required to achieve a topologically consistent river network (*i.e*. properly chained from sources to sea outlets) across different countries. For example, river discontinuities at international boundaries caused by differences among national GIS layers were resolved through extensive manual editing.

### River Network Attributes (RNA table)

In addition to the geographical information compiled in the RN table, physical attributes at the river segment level are needed to predict eel distribution and abundance using the EDA model (Tables 2, 3, 4). The RNA table includes altitude, temperature, water surface, cumulated variables calculated from the river outlet upstream to each river segment (e.g. cumulated height of dams, cumulated number of dams, and distance to the sea from each estuary to each river segment), cumulated variables calculated from the source downstream (e.g. upstream basin surface, Strahler and Shreve ranks), as well as discharge and seasonality (*i.e*. whether a river is permanent or seasonal). Finally, a coastal spatial gradient was introduced using a continuous variable: the distance to Gibraltar. Calculation of these variables is detailed below.

**Table 2:** Name and description of the physical attributes recorded for each river segment in France and the Iberian Peninsula, stored in the River Network Attributes (RNA) table (part 1).

Name	Description
Altitude of the river segment (m) (*altitudem*)	Altitude at which the river segment is located (m). In France, it is obtained from the Theoretical Hydrographic Network (RHT)^23^. In the Iberian Peninsula, it is computed at the centroid of each river segment using the European Digital Elevation Model (EU-DEM), version 1.1.^60,61^.
Distance to the sea (m) (*distanceseam*)	Distance from a river segment to the sea (m), calculated as the sum of the lengths of the segments and all downstream segments along the river network to the sea.
Distance to the source (m) (*distancesourcem*)	Distance to the farthest upstream source of a river segment (m), calculated as the sum of the lengths of the river segments (m) along the chain connecting that source to the current river segment. The distance includes the length of the current segment.
Cumulated number of dams (*cumnbdam*)	Cumulated number of dams between the sea and the river segment including obstacles located on the current river segment (see the Inventory of obstacles paragraph for data sources).
Cumulated height of dams (m) (*cumheightdam*)	Cumulated height of dams (m) between the sea and the river segment, including obstacles located on the current river segment. The cumulated height corresponds to the sum of dam heights, without predictions for missing values (see the Inventory of obstacles paragraph for details on the different fields available).
Total surface of the unit basin (m^2^) (*surfaceunitbvm*2)	Total surface of the unit basin, including land and aquatic surfaces (m^2^), corresponding to one river segment. A catchment is split into unit catchment surrounding river segments. In France, unit basin surfaces are derived from the RHT^23^. In the Iberian Peninsula, land surface was computed from INSPIRE networks for Spanish, Portuguese, and international basins.
Upstream basin surface (m^2^) (*surfacebvm*2)	Land surface of the basin located upstream from the river segment (m^2^), including the unit basin of the segment. In France, this variable is derived from the RHT^23^. In Spain, Portugal, and international basins, upstream basin surfaces were computed using routing algorithms developed within the SUDOANG project.
Strahler rank (*strahler*)	Order assigned to each river segment based on the hierarchy structure of the river network. As different national networks have different spatial resolution, Strahler ranks must be interpreted separately for each country.
Shreve rank (*shreve*)	Total number of upstream sources contributing to a river segment. All source river segments are assigned an order of one. Starting at those headwaters, numbers are added at the confluence of each river^45^. As different national networks have different spatial resolution, Shreve ranks must be interpreted separately for each country.
Sea code (*codesea*)	Code indicating the sea into which the river flows: “A” for Atlantic, “M” for the Mediterranean.
Name (*name*)	Name of the river. In France, it corresponds to the name of the hydrographic subsector from the “Database on Thematic Cartography of the Water Agencies and the Ministry of the Environment” (BD Carthage), *lbsoussect*, having the largest spatial intersection with the river segment; the corresponding *codesoussect* (hydrographic subsector code) is also provided in the BD Carthage database. In Spain, it corresponds to the *nom*_*rio* field from “Tramos de ríos de España clasificados según Pfafstetter modificado”^31^. In Portugal, it corresponds to the *nome* field from the “Rede hidrografica GeoCodificada” layer^32^.
Pfafstetter of the river (*pfafriver*)	Spain: Code of the main river according to the Pfafstetter hierarchical coding system^62^, corresponding to the *pfafrio* field from the “Tramos de ríos de España clasificados según Pfafstetter modificado” layer^31^. This information is not available for France and Portugal.
Pfafstetter of the river segment (*pfafsegment*)	Spain: Code of the river segment according to the Pfafstetter hierarchical coding system^62^, corresponding to the *pfafsegment* field from “Tramos de ríos de España clasificados según Pfafstetter modificado” layer^31^. This information is not available for France and Portugal.

**Table 3:** Name and description of the physical attributes recorded for each river segment in France and the Iberian Peninsula, stored in the River Network Attributes (RNA) table (continued, part 2).

Name	Description
Basin (*basin*)	Name of the basin. In France, it corresponds to the name of the hydrographic sector *lbsecteurh* from the “Database on Thematic Cartography of the Water Agencies and the Ministry of the Environment” (BD Carthage), selected as the sector having the largest spatial intersection with the river segment; the corresponding hydrographic sector code (*codesect*) is also provided in the BD Carthage^33^. In Spain, the basin corresponds to the *basin* field from the “Tramos de ríos de España clasificados según Pfafstetter modificado” layer^31^; this field might not always be consistent with hydrographic basins, *i.e*., segments with the same sea *idsegment* might have several basin names. In Portugal, the basin corresponds to the *name* field from the “Rede hidrografica GeoCodificada” layer^32^.
River width (m) (*riverwidthm*)	In France, the river width corresponds to the width of the river in “natural” conditions^37^. In the Iberian Peninsula, the river width is derived from two sources. For the largest rivers and reservoirs, river width is derived from the MERIT Hydro raster dataset^25^. For the remaining segments, the river width has been calculated using a model based on drainage area, basins grouped by runoff categories, and calibrated on data comprising both randomly collected width measurements and electrofishing data.
River width data source (*riverwidthmsource*)	Source used to derive river width. In France, it corresponds to the 2019 update of the RHT width model^37^. In the Iberian Peninsula, this field indicates either no computation (for endorheic rivers), the MERIT Hydro raster dataset^25^, or the model used to predict river width.
Temperature (^∘^C) (*temperature*)	Mean air temperature derived from the CCM database^26^ based on the spatial intersection between the CCM unit basin and river segments. Temperatures in the CCM originate from the WORLDCLIM database, which provides interpolated climate surfaces for global land areas for to 1950-2000 reference period^63^.
Wetted surface of the river segment (m^2^) (*wettedsurfacem*2)	Wetted surface of a river segment (m^2^), calculated as riverwidthm*lengthriverm, except when the river segment overlaps with other water bodies (*i.e*. when a river surface polygon covers a segment polyline, the overlapping part is excluded from the calculation). In France, this corresponds to the “theoretical channel surface” under “natural” conditions. Non-overlapping water surfaces are reported in the *wettedsurfaceotherm*2, while the *waterbody*_*unitbvwater* surface is also available for download for further details^18,19^. In all three countries, river networks have been simplified so that no bifurcations or islands are present.
Wetted surface of other water bodies (m^2^) (*wettedsurfaceotherm*2)	Wetted surface (m^2^) of the water bodies within the unit basin. In France, different water bodies such as canals, rivers, lakes, reservoirs, and lagoons have been split per unit basin (source: BD TOPO© Hydrographie^24^); only permanent water surfaces corresponding to lagoons, estuaries, natural flows, channels, reservoir-dams, reservoir-bassins, marshes, lakes, reservoirs are considered. In Spain, water bodies are derived from “Masas de agua superficial (polígonos) PHC 2015-2021”^39^, including estuaries, reservoirs, temporary lakes, river polygons, lakes, lagoons and coastal waters. In Portugal, water surfaces are derived from river water bodies, transitional water bodies and lagoons^41^ and reservoirs^42^, corresponding to estuaries, rivers, reservoirs, lagoons and lakes. The proportion of the length of each river segment not overlapping water body polygons has been computed for each river segment. As a consequence, the total aquatic surface within the unit basin corresponds to *wettedsurfaceotherm*2 + *wettedsurfacem*2.

**Table 4:** Name and description of the physical attributes recorded for each river segment in France and the Iberian Peninsula, stored in the River Network Attributes (RNA) table (continued, part 3).

Name	Description
River length (m) (*lengthriverm*)	Total length (m) of the river, measured along the river network from the farthest source to the upstream limit of the estuary.
Eel Management Unit (*emu*)	Eel Management Unit (EMU) according to regulation No 1100/2007 of 18 September 2007 establishing measures for the recovery of the European eel stock. In France, the EMU corresponds to the river basin district. In Spain, the EMU corresponds to administrative regions (Autonomous Communities). The whole territory of Portugal constitutes a single EMU, and there is a transboundary EMU in the Minho estuary. Spanish and Portuguese EMUs do not correspond to hydrographic regions, so reporting per EMU does not correspond to basin production.
Slope of the river segment ($\frac{{\rm{m}}}{{\rm{m}}}$) (*slope*)	Slope expressed as the elevation change divided by horizontal distance (rise/run, in $\frac{{\rm{m}}}{{\rm{m}}}$). In France, this corresponds to the slope calculated along the river segment geometry (*i.e.* along the river arc) as provided in the RHT, divided by 1000^23^. In Spain, slope is calculated at the unit basin level as the difference between the maximum and minimum elevations observed within the unit basin ("Subcuencas de ríos completos clasificadas según Pfafstetter modificado”^31^), and therefore does not strictly represent the slope along the river course. In Portugal, slope corresponds to the mean terrain slope of the unit basin calculated within the HydroATLAS project^27^ converted from degrees to $\frac{{\rm{m}}}{{\rm{m}}}$. Projection of RiverATLAS variables onto the SUDOANG network was performed using a 500 m buffer and excluding river segments with Shreve ranks ≤ 2 to avoid assigning slope values from small streams to larger river segments; consequently, slope is not available for some river segments.
Natural discharge ($\frac{{{\rm{m}}}^{3}}{{\rm{s}}}$) (from RiverATLAS) (*dis*_*m*3_*pyr*_*riveratlas*)	Annual average natural discharge derived from the WaterGAP v2.2 (data of 2014)^47^ as provided in the RiverATLAS dataset^27^. The same spatial projection method as for slope was applied; as a consequence, discharge values are only available for river segments where a reliable projection from RiverATLAS was possible, and not all segments have an associated value.
Natural minimum discharge ($\frac{{{\rm{m}}}^{3}}{{\rm{s}}}$) (from RiverATLAS) (*dis*_*m*3_*pmn*_*riveratlas*)	Annual minimum natural discharge derived from WaterGAP v2.2^47^, following the same projection approach as for the natural discharge.
Natural maximum discharge ($\frac{{{\rm{m}}}^{3}}{{\rm{s}}}$) (from RiverATLAS) (*dis*_*m*3_*pmx*_*riveratlas*)	Annual maximum natural discharge derived from WaterGAP v2.2^47^, following the same projection approach as for the mean discharge.
Median natural discharge ($\frac{{{\rm{m}}}^{3}}{{\rm{s}}}$) (*module*)	In France, median natural discharge (interannual module) calculated on the RHT^37^. In the Iberian Peninsula, this variable corresponds to the *dis*_*m*3_*pyr*_*riveratlas* attribute, note that it is not available for all river segments.
Risk of drought (*drought*)	Categorical variable describing the temporary flow status of river segments. Rivers are classified into five classes: (1) permanent, (2) seasonal, (3) intermittent, (4) ephemeral, and (5) not classified (no information available). In practice, river segments classified as intermittent (3) or ephemeral (4) are assumed not to provide suitable habitat for eels in model predictions, while segments with no data (class 5) are, by default, treated as permanent rivers (*i.e.* flowing year-round). In the Mediterranean basin of Spain, drought risk and river classification were assessed by the University of Córdoba and are available in the *drought*_*type*_*calc* field. In northern Portugal, rivers with minimum discharge lower than 0.1 m^3^s^−1^ are classified as intermittent (class 3), whereas in the southern Iberian Peninsula this threshold is 0.3 m^3^s^−1^. The upstream limit of flowing rivers remains uncertain in many cases, reflecting the limited availability of harmonised datasets on river intermittency.
Source and rules of temporary river class assignment (*drought*_*type*_*calc*)	Details about the source of data for drought calculation.

#### River width

This variable was required to estimate the water surface area of each river segment, which is key for eel density and biomass calculations within the EDA framework. In France, we collected river width from published model-based estimates^37^. This model is built on river reaches with unaltered morphology (e.g. not affected by channelization, reshaping or embankment) so the estimated width represents an approximation of “natural” river width. We directly used the published outputs without additional processing. In the Iberian Peninsula, we obtained river width from two complementary sources depending on river size and data availability:

- For the largest rivers and reservoirs, we derived river width from the MERIT Hydro^25^ raster dataset (license CC-BY-NC 4.0), which provides globally consistent gridded estimates of river channel width. For each river segment, we projected the MERIT Hydro raster onto 10 points evenly distributed along the segment and calculated the mean of the corresponding raster width values. This projection excludes low Strahler orders. Because the fluvial network is represented at a finer spatial resolution in Spain than in Portugal, we excluded river segments with Strahler orders 1 to 3 in Spain, and Strahler order 1 in Portugal, retaining only the width values associated with reservoirs and main river segments. This approach avoided attributing unrealistically large width values to downstream sections of small tributaries intersecting reservoirs^38^.
- For the remaining river segments, we estimated river width using a model based on drainage area, with basins grouped by runoff categories, and calibrated using a dataset combining randomly collected river width measurements and electrofishing survey data^38^ (Table 3).

#### Water surface

To obtain the number of eels, we have to multiply the estimated densities (biomass of eels per unit surface area, estimated by EDA) of yellow and silver eels by the water surface area (Table 3). We distinguish between rivers represented in GIS as line features and other aquatic habitats or water bodies represented as polygons (including, in some cases, large rivers). For rivers, we calculated river water surface by multiplying river segment length (Table 1) by the estimated river channel width (Table 3). For adjacent water bodies (*i.e.* estuaries, lagoons, reservoirs, lakes, and temporary lakes), we have retrieved water surface information from BD TOPO® in France^24^ and from the official web portals of the corresponding authorities in Spain^39^ and Portugal^40–42^ (Table 3). In the case of estuaries, for the Iberian Peninsula, we obtained water surface area and habitat type from the Water Information System for Europe (WISE) transitional waters. However, in France, the downstream part of some estuaries is not yet included in our database. This is due to the time-consuming nature of the spatial processing, which requires manual editing of spatial layers to remove overlaps.

We used GIS tools to split river water surfaces among river reach unit basins, so that, for instance, a large lake polygon was split into several smaller sub-polygons following the unit basin boundaries. The total surface area of these split polygons (lakes, reservoirs, etc.) was then used to calculate the water surface area of adjacent water bodies within each unit basin^43^. As polygon layers describing adjacent water bodies overlap with those of the rivers, summing both surfaces would have resulted in an overestimation of the surface area. For this reason, in those cases, we considered only the surface area of adjacent water bodies and the overlapping river water surface was removed from the calculations. Finally, the total aquatic habitat surface of each river reach was calculated as the sum of the segment surface and the surface of adjacent water bodies (Table 3).

#### Variables calculated from river network topology

We developed routing algorithms^44^ to estimate cumulated metrics, including upstream basin surface area, distance to the sea, distance to the source, Shreve^45^ and Strahler^46^ ranks by taking a given river segment as the starting point (Table 2).

#### Discharge

For Spain and Portugal, we downloaded river discharge data from RiverATLAS^27^, and projected minimum, maximum and average natural discharge from WaterGAP v2.2^47^. Because the RiverATLAS river network differs from the national river network used in this study, discharge values were transferred using a 500 m buffer, by excluding small tributaries (Shreve rank less than or equal to 2) and matching river segments based on the nearest Strahler rank. In France, we obtained median discharge derived from a hydrological model based on the RHT network^37^. In both cases, discharge corresponds to “natural” conditions, *i.e*., not accounting for human water abstraction or flow regulation (Table 4). We used this attribute to calculate the risk of a river being temporary.

#### Temporary status of rivers

The temporary flow status of rivers is required to determine whether a given river segment can be considered an available habitat for eels. Based on flow presence, rivers were classified into five categories according to the hydromorphological characterisation established by the Spanish government under the Order ARM/2656/2008:

1. Permanent: under natural conditions, the watercourse has flowing water throughout the year.
2. Seasonal: under natural conditions, the watercourse exhibits marked seasonality, characterised by low flows or dry sections in summer. Flow is present for an average period of at least 300 days per year.
3. Intermittent: under natural conditions, the watercourse is highly seasonal, with water flowing for an average period of 100-300 days per year.
4. Ephemeral: under natural conditions, surface water flows only sporadically, typically following storm events.
5. Not classified: no information is available on the temporary flow status of the river.

We considered that temporary rivers corresponding to the categories “intermittent” or “ephemeral”, as well as rivers located upstream of dried sections, are no longer available habitat for eels. In contrast, “permanent” and “seasonal” rivers were regarded as potential eel habitats. This led to the removal of a very large number of headwaters and reservoirs as potential eel habitat in the south of the Iberian Peninsula. In Spain, information on river temporary status was initially provided by the Spanish Ministry for Ecological Transition (MITECO), although with only partial spatial coverage. However, additional checks using satellite imagery showed that some rivers dry out during summer, especially in southern Spain. In these cases, given that many headwaters (below Shreve rank six) do not correspond to actual riverbeds when inspected using digital orthophotography and satellite imagery, flow data were used to calculate the risk of a river being intermittent. In Portugal, no data on intermittent rivers were available. However, river layers are less detailed than in Spain, therefore do not include many small rivers with high Shreve rank that are more susceptible to drying out. As a consequence, the risk of including seasonal rivers was lower than in Spain. To estimate the risk of a river being intermittent, we used flow data. Rivers were classified as intermittent when no flow could be projected from RiverATLAS (NULL values) and when the Shreve rank was lower than six. Based on an analysis of the existing river classification and validation using satellite imagery, we classified rivers with a minimum discharge lower than 0.1 m^3^s^−1^ as intermittent in northern Portugal. Using a similar validation approach, this threshold was set to 0.3 m^3^s^−1^ in the southern Iberian Peninsula. In France, information on dry status was only available as point time series derived from gauging stations and was therefore not integrated into the spatial dataset. However, the RHT source dataset excludes many small rivers that are more susceptible to drying out. Consequently, no additional processing was required to remove this type of river from the SUDOANG dataset (Table 4).

**Fig. 1:**
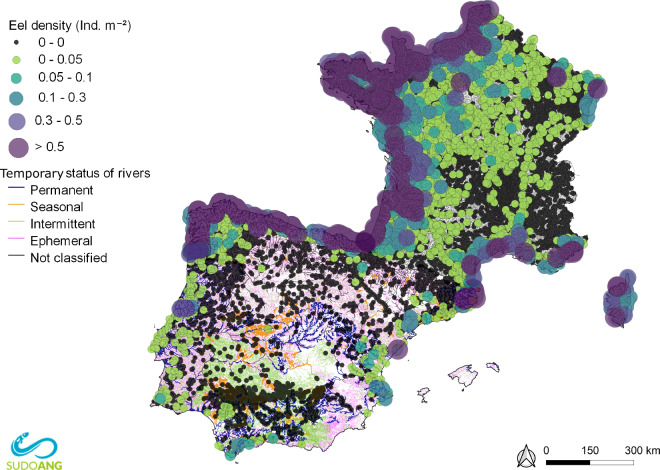
Spatial distribution of eel density (Ind. *m*^−^2) estimated from electrofishing surveys and the temporary flow status of rivers.

To our knowledge, this is the first attempt to build an international database of temporary rivers (Fig. 1). As such, it should be interpreted with caution. Developing a comprehensive database of rivers and their status across the entire Iberian Peninsula and France remains a challenge for the future (Table 4).

#### Distance to Gibraltar

**Fig. 2:**
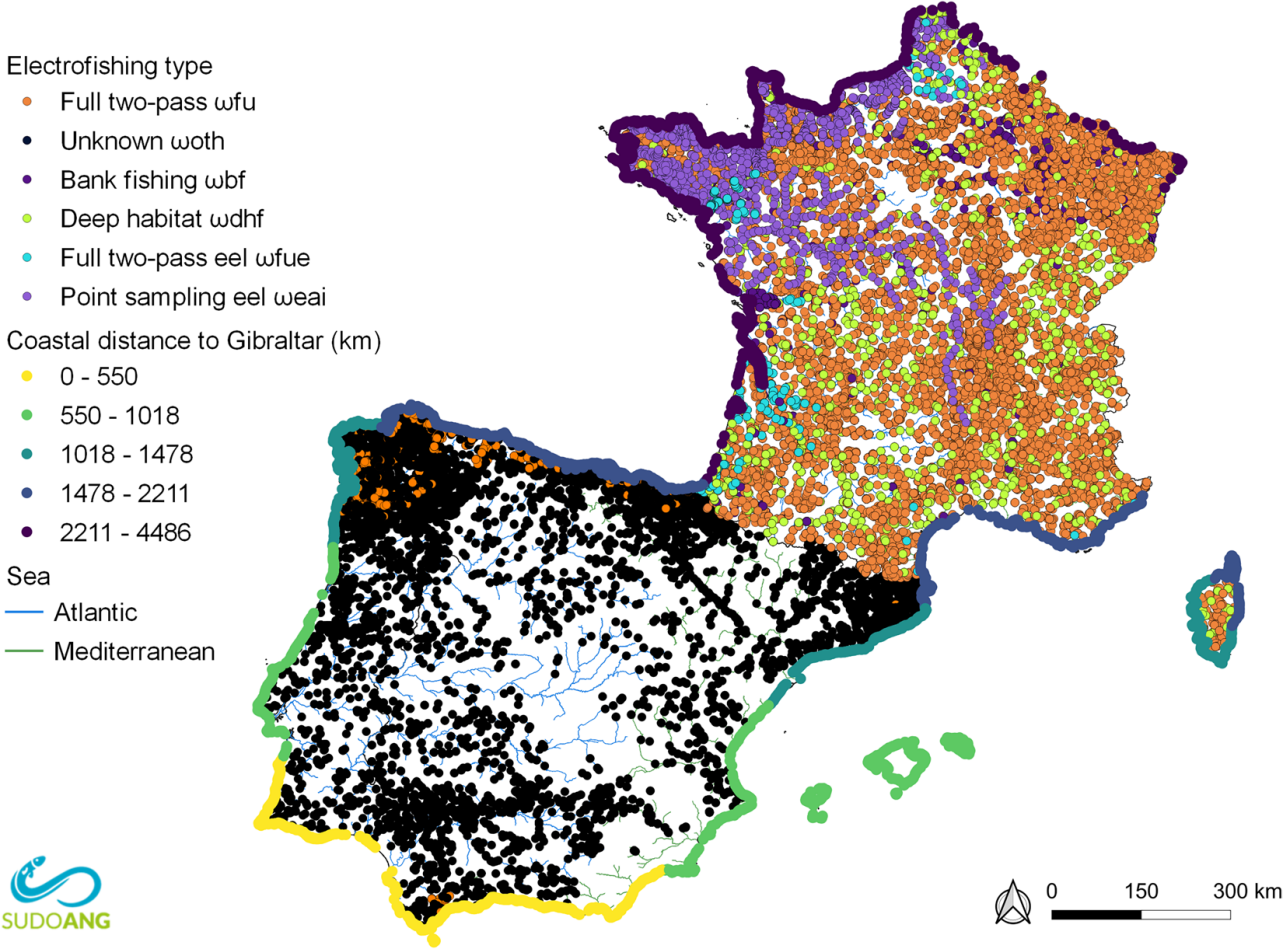
Location of electrofishing stations, categorized by type. The sampling protocol variable (*ω*) describes the different types of electrofishing protocols used in France. Eel-specific surveys conducted on index rivers are also indicated (*ω*_*eai*_, *ω*_*fue*_ see also Table 11). In the Iberian Peninsula, the type of electrofishing used could not be specified in detail, but surveys mostly correspond to single-pass or multi-pass electrofishing. In the absence of specific information, the type was set to an unknown *ω*_*oth*_. In Spain, all electrofishing surveys ing eels in the second pass are considered as full two-pass electrofishing *ω*_*fu*_. The coastal distance to Gibraltar (in km) is indicated for each river mouth; values are negative in the Mediterranean. The coulour of the main rivers varies according to the sea they meet.

Distance to Gibraltar is defined as the length of the coastline between Gibraltar and river mouths, excluding the coastlines of estuaries and bays. Distances were assigned positive values in the Atlantic and negative values in the Mediterranean. This metric provides a continuous variable that complements the categorical variables of country, area, and basin, allowing a clear separation between Atlantic and Mediterranean systems (Table 5) (Fig. 2).

**Table 5:** Name and description of the topological and hydrographical characteristics recorded for each watershed in France and in the Iberian Peninsula, stored in the River Network (RN) watershed table.

Name	Description
Identifier of the sea segment (*seaidsegment*)	Identifier of the most downstream river segment when the river segment is flowing into the estuary to meet the sea.
Geometry of the basin polygon (*geom*)	The geometry (*i.e*. PostGIS spatial data type used to represent spatial objects) of the basin, using the coordinate system EPSG:3035. This geometry is used to represent the spatial component of geographic features in a planar coordinate system. The type is a POLYGON.
3 zones for recruitment estimation in GEREM model (*gerem*_*zone*_3)	Three geographic areas along the coast where identical recruitment is expected in the 3-zone configuration of the GEREM model.
4 zones for recruitment estimation in GEREM model (*gerem*_*zone*_4)	Four geographic areas along the coast where identical recruitment is expected in the 4-zone configuration of the GEREM model. These areas are selected for the GEREM and EDA model (corresponding to the SUDOE recruitment zones used to predict *delta* and *gamma* in Table 5).
5 zones for recruitment estimation in GEREM model (*gerem*_*zone*_5)	Five geographic areas along the coast where identical recruitment is expected in the 5-zone configuration of the GEREM model.
CCM Identifier (*ccm*_*wso*_*id*)	CCM Identifier of the river basin to which the main river belongs, collected from the seaoutlet table of the CCM database.
Country code (*country*)	Code of the country where the basin belongs: Spain (SP), France (FR) and Portugal (PT).
Eel Management Unit (*emu*_*name*_*short*)	Eel Management Unit (EMU) according to regulation No 1100/2007 of 18 September 2007 establishing measures for the recovery of the European eel stock. In France, the EMU corresponds to the river basin district. In Spain, the EMU corresponds to administrative regions (Autonomous Communities). The whole territory of Portugal constitutes a single EMU, and there is a transboundary EMU in the Minho estuary. Spanish and Portuguese EMUs do not correspond to hydrographic regions, so reporting per EMU does not correspond to basin production.
Geometry of the river mouth point (*geom*_*outlet*)	The geometry (*i.e*. PostGIS spatial data type used to represent spatial objects) of the river mouth, using the coordinate system EPSG:3035, type POINT.
Name of the river basin (*name*_*basin*)	Name of the river basin, collected from the seaoutlet table of the CCM database.
Coastal distance to Gibraltar (in km) (*dist*_*from*_*gibraltar*_*km*)	Coastal distance to Gibraltar (in km) computed along the coastline, positive on the Atlantic coast and the channel and negative in the Mediterranean.
Coast code (*name*_*coast*)	Code of the coast where the river flows into the estuary to meet the sea: Atlantic Spain (ATL_SP), Atlantic Portugal (ATL_PT), Cantabrian Sea (Cant), Mediterranean Spain (Med_SP), Mediterranean France (Med_FR), Atlantic France (ATL_FR).

### River Network Eel attributes (RNE table)

The RNE table compiles estimates of eel abundance and their distribution characteristics in France and the Iberian Peninsula, obtained through the implementation of the EDA 2.3 model^48^. The RNE table provides predictions at the river segment level of:

- The probability of eel presence, density (Ind. m^−2^), number and size structure (Table 6).

**Table 6:** Name and description of the eel abundance and biometric distribution variables estimated for each river segment in France and the Iberian Peninsula, stored in the River Network: Eel attributes (RNE) table (part 1).

Name	Description
Probability of presence of eel $\widehat{\Delta }$ (*delta*)	Prediction of the EDA delta model ($\widehat{\Delta }$) provided for each river segment. This is a GAM binomial model to predict presence / absence of eel. The delta model is based on 21 706 electrofishing data points in France and the Iberian Peninsula. The model predicts eel presence according to the following variables: (1) SUDOE recruitment zone (zone for recruitment defined by the project stakeholders during a workshop in Sukarrieta), (2) country, (3) fishing method, (4) year (factor) per SUDOE recruitment zone, (5) electrofishing station, (6) wetted area, (7) altitude, (8) distance from Gibraltar, (9) drainage density (*m*^−1^), which is defined as the sum of the length of segments of the basin located at a lower or equivalent distance of the sea, divided by the sum of the surface of the unit basins of those segments. Drainage density quantifies the ramification of a river catchment^64^: $\frac{\sum segment\,length}{\sum surface\,basin}$, (10) Downstream drainage wetted surface (an indicator of the presence of habitat in the river basin downstream): $\frac{\sum wetted\,surface\,riversegments+wetted\,surface\,other}{\sum surface\,basin}$
Density when eel is present $\widehat{\Gamma }$ (Ind. m^−2^) (*gamma*)	Prediction of the EDA gamma model ($\widehat{\Gamma }$) of eel abundance. This is a GAM model computed on a subset of the electrofishing dataset where observed eel densities are strictly positive. The model is predicted according to the following variables: (1) distance to the sea, (2) fishing method, (3) altitude, (4) year (factor) per SUDOE recruitment zone, (5) distance from Gibraltar, (6) interaction between cumulated height of dams and distance to the sea, (7) hydraulic density (m^−1^), (8) SUDOE recruitment zone, (9) cumulated height of dams and, (10) the interaction between altitude and distance to the sea. The gamma model is applied to all river segments and is not conditioned on any threshold value of the delta (probability of presence).
Eel density (Ind. m^−2^) (*density*)	Predicted eel density (Ind. m^−2^) for each river segment. The prediction is obtained by multiplying the delta and gamma components of the EDA model: $\widehat{d}=\widehat{\Delta }\widehat{\Gamma }$. This metric represents the eel standing stock and therefore includes both yellow and silver eels.
Number of eels (*neel*)	Number of eels predicted by the model for the river segment and its associated water bodies. It corresponds to the predicted eel density multiplied by the wetted surface of river segments *S*_*r*_ and the wetted surface of other water bodies *S*_*o*_ within the same unit basin: $\widehat{N}=\widehat{d}{S}_{r}+\widehat{d}{S}_{o}$. This metric represents the eel standing stock and therefore includes both yellow and silver eels.
Biomass of eels (kg) (*beel*)	Biomass of eels (kg) predicted by the model for each river segment and its associated water bodies. It is calculated by multiplying the number of eels in each size class (<150, 150-300, 300-450, 450-600, 600-750, >750) by the average weight of eel in this size class in the corresponding country (see tables 3.6 and 5.11 in EDA report^48^). This metric represents the eel standing stock and therefore includes both yellow and silver eels.
Proportion of eel of size <150, 150-300, 300-450, 450-600, 600-750 and >750 mm (*peel*150, *peel*150300, . . . , *peel*750)	Proportion of eel in each size class within a river segment. Eels correspond to the eel standing stock and therefore include both yellow and silver eels that will migrate to the sea during the year. *p*_<150_ + *p*_150−300_ + *p*_300−450_ + *p*_450−600_ + *p*_600−750_ + *p*_>750_ = 1. The eels of class <150 mm and 150-300 mm are those that initially colonize the basin. Fewer large eels migrate upstream, but they might continue to colonize the basin. The eels of class 300-450 mm are the most abundant in number, they may mature both as male or female silver eels. Class 450-600 mm is the first class of eel that will produce mostly females. Eels larger than 600mm are large eel producing large female silver eels. The eels of class >750 mm are the largest. They have a high probability to mature, and have the largest fecundity.
- The proportion of eel reaching the silver stage by sex, as well as predicted silver eel proportions by size class and sex (Table 7).

**Table 7:** Name and description of the eel abundance and biometric distribution variables estimated for each river segment in France and the Iberian Peninsula, stored in the River Network: Eel attributes (RNE) table (part 2, regarding silver eels).

Name	Description
Number of silver eels (*nsilver*)	Number of silver eels estimated by the model for the river segment and associated water bodies. This number is calculated from the number of eels, broken down per size class and from the silver eel model which predicts the proportion of silver male and female eel on the river segment per size class. The number of silver eels corresponds to the sum of the number of silver eels per size class.
Biomass of silver eels (male + female) (kg) (*bsilver*)	Biomass of silver eels (kg) estimated by the model on the river segment and associated water bodies. It is calculated by multiplying the number of silver eels in each size class by the average weight of silver eels in this size class and then summing over size class in the corresponding country.
Proportion of silver eel of size classes 150-300, 300-450, 450-600, 600-750 and >750 mm (*psilver*150300, …, *psilver*750)	Proportion of silver eels estimated from each size class in a river segment. Silver eels of size class 150-300 mm can only be male, while size class 300-450 mm are mostly male. 450-600 mm class are mostly female. Sizes 600-750 mm and >750 mm are always female.
Proportion of silvering (*psilver*)	Proportion of eels that undertake silvering each year.
Proportion of male of size classes 150-300, 300-450, 450-600 mm (*pmale*150300, …, *pmale*450600)	Proportion of males at each size class of 150-300, 300-450 and 450-600 mm that will undergo a silvering process during the year in a river segment. This proportion is the product of the proportion of eel in this class as predicted by the size structure multinomial model, multiplied by the proportion of silver in this class predicted by the silvering multinomial model.
Proportion of female of size classes 300-450, 450-600, 600-750 and >750 mm (*pfemale*300450, …, *pfemale*750)	Proportion of females at each size class of 300-450, 450-600, 600-750 and >750 mm that will undergo a silvering process during the year in a river segment. This proportion is the product of the proportion of eel in this class as predicted by the size structure multinomial model, multiplied by the proportion of silver in this class predicted by the silvering multinomial model.
Proportion of males among silver eel (*pmale*)	Proportion of males among silver eels predicted by the model. To avoid confusion, the proportion from the model (yellow/male/female) has been corrected to only account for silver eels. So: *pmale* = 1 − *pfemale*
Male sex ratio (*sex*_*ratio*)	Ratio of silver male versus females in the predicted population of each river segment: $\frac{{N}_{male}}{{N}_{female}}$.
- The cumulated number and the size structure of silver eels emigrating from upstream river segments (Table 8). This information is particularly valuable for assessing the impact of hydropower equipment, for which the size structure is one of the main predictors of mortality risk during turbine passage^49^.

**Table 8:** Name and description of the eel abundance and biometric distribution variables estimated for each river segment in France and the Iberian Peninsula, stored in the River Network: Eel attributes (RNE) table (part 3, regarding upstream silver eel calculations).

Name	Description
∑_*upstream*_*female*_300−450_ (*cnfemale*300450), ∑_*upstream*_*female*_450−600_ (*cnfemale*450600), ∑_*upstream*_*female*_600−750_ (*cnfemale*600750), ∑_*upstream*_*female*_>750_ (*cnfemale*750)	Cumulated number of silver eel females per size class estimated by the model in the basin located upstream from the current river segment.
∑_*upstream*_*male*_150−300_ (*cnmale*150300), ∑_*upstream*_*male*_300−450_ (*cnmale*300450), ∑_*upstream*_*male*_450−600_ (*cnmale*450600)	Cumulated number of silver eel males per size class estimated in the basin located upstream from the current river segment.
∑_*upstream*_*silver*_150−300_ (*cnsilver*150300), ∑_*upstream*_*silver*_300−450_ (*cnsilver*300450), ∑_*upstream*_*silver*_450−600_ (*cnsilver*450600), ∑_*upstream*_*silver*_600−750_ (*cnsilver*600750), ∑_*upstream*_*silver*_>750_ (*cnsilver*750)	Cumulated number of silver eels per size class estimated in the basin located upstream from the current river segment.
∑_*upstream*_∑_*τ*_*silver*_*τ*_	Cumulated number of silver eels estimated in the basin located upstream from the current river segment.

The EDA 2.3 model extrapolates the results of a model based on electrofishing carried out only in freshwater habitats. The results are extrapolated to all habitats, including lagoons and estuaries, where this extrapolation remains uncertain.

### Inventory of obstacles

This dataset^22^ contains information on obstacles collected by the SUDOANG project. This information, together with the attributes included in the RN and RNA tables, allows the estimation of the cumulated impact of obstacles along the river network (*i.e*. using river paths defined in the SUDOANG river network (RN table), and altitude information from the RNA table). Artificial obstacles are classified into 10 types according to the Adaptive Management of Barriers in European Rivers (AMBER) project^50^. Additional types (e.g. penstock pipes) were included to account for obstacles reported in national databases that do not fit the AMBER classification. In some cases, dams located on different distributaries are connected, forming dam-networks. In those cases, we retained only the dam(s) located on the main river course, based on a hierarchical classification of dams. In France, obstacles are compiled from three pre-existing databases: the Referential of Flow Obstacles (Referentiel des Obstacles à l’Ecoulement)^28^, the Information on Ecological Continuity (ICE)^29^, and the Flow Obstruction Database (Base de Données des Obstacles à l’Ecoulement, BDOE). The data integrated into the SUDOANG 1.0.4 database correspond to a database dump from 12 September 2019. In Spain, obstacle data were obtained from the MITECO Ministry, the Basque Water Agency (URA), the Catalan Water Agency (ACA), the University of Girona, the University of Córdoba, the Xunta of Galicia, and the AMBER project. In Portugal, data were obtained from the Portuguese Water Agency (APA), the University of Lisbon, the University of Porto and the AMBER project. We selected only obstacles that are currently standing, *i.e*. we removed those that were planned, under construction, or destroyed. Dikes, longitudinal control structures and grates were also excluded Table 9.

**Table 9:** Obstacle classification according to the data collected in the AMBER project.

Name	Description
Bridge	A structure that is built over a river to allow people or vehicles to cross.
Culvert	A tunnel or pipe carrying a river or open drain under a road or railway.
Dike	An embankment used to hold back water.
Dam	Structure that blocks the river and extends across the river bed to the flood plain.
Ford	A shallow crossing-place in a river.
Penstock pipe	Group of pipes that transport pressurised water from a reservoir (dam) to the turbines installed in a hydro-electric power plant.
Rock ramp	A weir made of rocks.
Weir	Structure across a river that does not extend to the flood plain.
Other	Structure that is not covered by previous definitions.
Unknown	Unknown.

Some eel passes included a passability assessment carried out by national experts. This information was incorporated into the inventory of obstacles as an attribute (Table 10).

**Table 10:** Expert judgement of eel pass efficiency by passage type for eel.

Name	Enhances passage for eel
Pool pass	Yes
Channel	Yes
Eel pass	Yes
Rock ramp	Yes
Denil type pass	No
Other	No
Fish lift	No

We projected obstacles onto the SUDOANG river network by assigning them to the nearest river segment within 300 m distance. To avoid misplacing large obstacles, particularly in southwestern France, SUDOANG experts reviewed and manually corrected obstacle locations where necessary. In France, we also applied an algorithm to extract the most reliable obstacle height information from the three available databases. In the Iberian Peninsula, data providers validated and corrected obstacle locations and heights using a dedicated Shiny application developed by us, which allowed them to directly edit locations and obstacle height values. Finally, we built a table containing approximately six million records, relating each river segment to all obstacles located downstream of that segment. While each obstacle appears multiple times (once per upstream segment), this table enables efficient computation of cumulated obstacle indicators, such as the cumulated height of dams, for all river segments.

We used a Generalized Linear Model (GLM) to estimate missing obstacle height data (log transformed height; *family = Gaussian, link = identity*). In France, the model was fitted using river segment slope, median river discharge, and hydrographic basin as predictors^51^. Obstacle type was included through interaction terms with all predictors:

$$log(h) \sim log(medianflowm3ps+1)\ast obstacle\,\_type+log(slope+1)\ast obstacle\_type+basin\ast obstacle\_\,type$$

In the Iberian Peninsula, we implemented a simpler model based on obstacle type^52^, as information on river discharge or slope was not available for all river segments.

$$log(h) \sim obstacle\_type$$

Tests conducted in France showed that including additional factors such as the presence of a fish pass and eel passability^51^ did not improve model performance^44^. Although more complex models could be tested in France due to higher data availability, they were not retained in the final workflow. To ensure methodological consistency among countries, and because this type of information was too limited in the Iberian Peninsula, we used only dam height to model the cumulative height of obstacles for each river segment.

### Electrofishing data

We projected eel densities and biometries derived from electrofishing surveys onto all river segments of the SUDOANG river network (RN table) in France and the Iberian Peninsula. We collated electrofishing eel records from the Iberian Society of Ichthyology (SIBIC), the French Biodiversity Office (OFB) for two databases (ASPE and RSA), and 35 regional data providers in the Iberian Peninsula. We imported the collected data into the DataBase for EEL (DBEEL), a database designed to store eel data and developed during the POSE project^17^. Electrofishing data are structured hierarchically. The first level corresponds to the electrofishing station. We projected the stations onto the SUDOANG river network (RN table) using the nearest river segment within a distance of 300 m, and we removed duplicates located less than 50 m apart from each other. The second level corresponds to the electrofishing operation, defined as a sampling event carried out at a given station on a specific date, during which station-level characteristics are recorded. We also deleted duplicate electrofishing operations with the same date^53^.

Electrofishing data originate from different monitoring programs applying different sampling protocols (Fig. 2). We therefore categorized electrofishing data into different types according to the protocol used in each country (Table 11). For electrofishing surveys with two or more passes, we applied Carle and Strub methodology^54^, as implemented in the FSA package in R^55,56^, to estimate eel densities, fishing efficiencies, and the sampled surface area of electrofishing stations. For one pass electrofishing samplings, we used the average regional electrofishing efficiency to estimate density. Estimated eel densities derived from electrofishing surveys are shown in Fig. 1, together with the temporary status of the rivers.

**Table 11:** Type of electrofishing for eel in France and the Iberian Peninsula. The number of operations by country is also indicated.

Code	Label	Description	Target species	Number of operations		
				France	Spain	Portugal
*ω*_*fu*_	Full two-pass	Two-pass electrofishing with prospection of the entire surface by foot.	All	20375	7	12017
*ω*_*oth*_	Unknown	Unknown electrofishing method.	All	0	862	1980
*ω*_*bf*_	Bank fishing	Electrofishing from the bank in deep wide streams, might be partially operated from a boat.	All	2993	0	0
*ω*_*dhf*_	Deep habitat	Electrofishing, following a specific point sampling method for deep habitat (boat)^65^.	All	5353	0	0
*ω*_*fue*_	Full two-pass eel	Full two-pass electrofishing by foot, but targeted on eel.	Eel	731	0	0
*ω*_*eai*_	Point sampling eel	Electrofishing, eel specific point sampling (by foot)^66^.	Eel	1829	0	0

When available, we imported individual measurements of eel length and weight into SUDOANG 1.0.4. Additionally, for silver eels, we collected eye diameter, pectoral fin length, presence of neuromasts along the lateral line, and body contrast. We reviewed biometric data, removed outliers, and ensured that the stage and sex were correctly assigned according to Durif’s criteria^48,57^. Raw electrofishing data^20^ and processed datasets used prior to modelling^21^ are provided via Zenodo.

## Data Records

The SUDOANG 1.0.4 database consists of three tables (the Eel Atlas: RN, RNA, and RNE) and is complemented by three additional datasets used as inputs to the EDA model and published separately:

- Eel Atlas (SUDOANG 1.0.4)^18^: this database is structured into three tables, the River Network (RN, Table 1), the River Network Attributes (RNA, Tables 2, 3, 4), and the River Network Eel (RNE, Tables 6, 7, 8). This atlas corresponds to a PostgreSQL database^19^ and is also available for download in other formats via the SUDOANG project website (https://sudoang.eu/en/).
- Electrofishing data for eel in the Iberian Peninsula^20^: this dataset compiles raw data on electrofishing for European eel collected in the Iberian Peninsula. Raw electrofishing data for France are already published (ASPE database^30^).
- Eel data (*Anguilla anguilla*) and associated environmental variables used to fit the EDA model in the SUDOE area^21^. We used this dataset to calibrate the EDA model in the three countries.
- Cumulated dam impact in France and the Iberian Peninsula^22^: this dataset includes both raw obstacle data (e.g. dam location and type) and modelled information describing cumulated dam impacts along river networks in the three countries.

Altogether, these datasets include 515011 river segments, 106400 obstacles belonging to nine types, and 23080 electrofishing stations. A total of 48719 electrofishing operations were conducted, of which 18827 sampled eels, resulting in 494163 individual eel records collected between 1985 and 2020 across the three countries. This information is disaggregated by country and marine coastline in Table 12.

**Table 12:** Summary of rivers, obstacles^22^ and electrofishing^21^ data, selected (after data cleaning) to feed the EDA model. Rivers corresponds to the RN (number and length of river segments) and RNA table (water surface)^19^.

		Atlantic			Mediterranean		E. Channel	North Sea
		France	Spain	Portugal	France	Spain	France	France
Rivers	Number of river segments	56187	201239	75056	26811	124160	25187	6371
	Length of river segments (km)	141908	310948	85230	62657	193856	60928	17847
	Water surface of rivers (*km*^2^)	534	919	509	291	589	225	73.8
Other water bodies detail	Total water surface of other water bodies (*km*^2^)	3060	3651	1486	2079	926	868	253
	Reservoirs (*km*^2^)	1566	2174	649	652	681	517	124
	Rivers and estuaries (*km*^2^)	1244	704	816	490	44	296	95
	Canals (*km*^2^)	43	0	0	40	0	48	29
	Lakes (*km*^2^)	207	211	5	342	71	6	4
	Marshes (*km*^2^)	0	557	0	3	1	1	1
	Lagoons (*km*^2^)	0	5	16	552	129	0	0
Obstacles	Bridges	3281	2627		1866	25	1130	1208
	Culverts	3161	4		1715	19	1136	1291
	Dams	4837	3944	364	1503	1063	942	930
	Fords	437	20		87	15	21	25
	Others	1776			1042		626	1305
	Penstock Pipes		34			31		
	Unknown	1020	2470	461	1128	1658	1573	1052
	Rock ramps	2361			2597		581	810
	Weirs	21132	6056	32	9258	2541	11254	3951
Electrofishing surveys	Number of stations	5956	7103	759	3125	1801	2683	1653
	Number of operations (one pass)	7486	1494	73	3370	796	4914	3372
	Number of operations (two passes and more)	7226	10525	896	3989	2284	2115	159
	Maximum number of passes	4	5	3	3	3	3	3
	Number of eels	198764	104157	5393	55190	3795	119784	7080

## Technical Validation

- River widths: we used river widths measured during electrofishing surveys to check that widths derived from the MERIT Hydro dataset for large rivers were of the order of magnitude. This was nearly always the case, except in river sections containing mid-channel islands.
- Temporary status: for the Mediterranean part of Spain, we verified the temporary flow status of all watersheds in collaboration with the University of Córdoba, using satellite imagery and local expert knowledge.
- Obstacles: in the Iberian Peninsula, we initially built the obstacle database using data provided by the Spanish Ministry for Ecological Transition (MITECO) and the AMBER project. At this stage, we considered that obstacles located within 0.01 m of each other corresponded to duplicate records (*i.e.* several structures listed at identical coordinates). Subsequently, when adding dams from other sources, we considered that dams located less than 500 m from an existing structure were duplicates. In France, as obstacle data originated from a single data provider, issues related to duplicate records were minimal. Nevertheless, we carried out additional checks based on dam height and structure name. We also screened out dams located at the outlets of small tributaries that had been incorrectly attributed to the main river course. We reviewed all dams higher than 100 m to verify that they were not penstock pipes. In addition, we validated the cumulated height of dams by comparing it with surrounding terrain elevation. When the cumulative dam height exceeded local elevation, we reviewed the height of each dam along the affected rivers using the Shiny validation application. Finally, we validated dam information through consultation with local experts.

## Usage Notes

The River Network (RN) table follows a hierarchical, tree-like structure, in which river segments are organised into parent-child relationships. The parent RN table is stored in the *dbeel_rivers* schema, while country-specific child tables are implemented in the *france*, *spain*, and *portugal* schemas, inheriting the same table structure from *dbeel_rivers*. This design allows complex GIS operations to be performed at the national level (*i.e.* within child tables), such as identifying river paths between two river segments within the same basin. At the same time, queries can be executed at the international level using the parent *dbeel_rivers* table, which is required to build and apply the EDA model across countries.

We created Spatial SQL functions that allow users to identify the river path between two river segments. These functions are stored in the function section of the Eel Atlas database^18^ (Table 1). In addition, we developed an algorithm in R^56^ to estimate the path to the sea, or the segments upstream from a given position in the watershed (*distance_sea* method^44^). Similarly, we implemented another R algorithm to estimate cumulated number and height of obstacles along river paths from the sea (*cumulated_dam_impact2* method^44^).

One of the limitations of the database when used at the international scale is that the spatial resolution of the hydrographic networks differs among countries. The Spanish river network is the most detailed, but it also included fictitious rivers in headwaters (e.g. derived from thalwegs based on elevation data and slope using GIS tools) that do not correspond to actual riverbeds. The Portuguese river network is less detailed and the French river network has the lowest spatial resolution. Because EDA predictions are based on wetted surface area, countries with more detailed river networks, characterised by higher Strahler and Shreve orders, tend to yield higher estimates of eel abundance. As a consequence, caution is required when comparing absolute eel biomass estimates between countries.

Another limitation of the database is related to the spatial representativeness of eel sampling. Current monitoring of eels relies primarily on electrofishing surveys, which are well adapted to small and shallow river systems but more difficult to implement in deep or large aquatic systems. Consequently, a part of the estimated eel abundance and biomass includes spatial extrapolation from sampled river habitats to other habitat types that are less frequently sampled, such as large rivers, reservoirs, lagoons, and estuaries. This issue of extrapolation also applies across countries and Eel Management Units. Sampling effort, and more specifically the availability of electrofishing data used in this exercise, is not uniform across the study area and remains very limited in some regions. This heterogeneity in data coverage may increase uncertainty in model-based estimates and underscores the need to reinforce and harmonise eel monitoring strategies among regions and habitat types. The development of complementary sampling approaches, such as environmental DNA or boat-based electrofishing in large systems^58^, could help address these limitations in future assessments.

## Data Availability

The database and datasets described in this Data Descriptor are publicly available via Zenodo, as cited in the Data Records section. This includes the Eel Atlas (SUDOANG 1.0.4) and the complementary datasets used as inputs to the EDA model, all of which are accessible through their corresponding DOIs.

## Code availability

All developed code is publicly available in GitLab repositories:

• Code to create and develop the database on rivers and their attributes: EDA_rivers^38^.

• Code to import eel records sampling by electrofishing and the inventory of obstacles into the SUDOANG database: EDA_dbeel^59^.

• Code to create and develop the EDA model by obtaining the eel predictions included in the RNE table: EDA_model^44^.
